# 4-[(4-Methyl­anilino)meth­yl]benzonitrile

**DOI:** 10.1107/S1600536811020757

**Published:** 2011-06-18

**Authors:** Bin Wei

**Affiliations:** a Ordered Matter Science Research Center, Southeast University, Nanjing 211189, People’s Republic of China

## Abstract

The asymmetric unit of the title compound, C_15_H_14_N_2_, contains two mol­ecules. The amine NH group connects two aromatic systems with similar C—N—C bond angles of 120.96 (16) and 119.75 (16)° for each independent mol­ecule. In contrast, the dihedral angles between the benzene rings are significantly different for the two independent mol­ecules, *viz*. 69.1 (2) and 79.9 (2)°.

## Related literature

For related literature on dielectric–ferroelectric materials (the title compound is a candidate for having good dielectric properties), see: Chen *et al.* (2010 ▶); Wang *et al.* (2005 ▶); Xiong *et al.* (2002 ▶); Ye *et al.* (2006 ▶); Zhao *et al.* (2008 ▶).
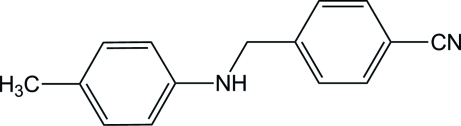


## Experimental

### 

#### Crystal data

C_15_H_14_N_2_
*M* *_r_* = 222.28
Monoclinic, 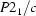

*a* = 12.485 (3) Å
*b* = 9.112 (3) Å
*c* = 22.180 (6) Å
β = 98.537 (6)°
*V* = 2495.3 (12) Å^3^
*Z* = 8
Mo *K*α radiation
μ = 0.07 mm^−1^
*T* = 293 K
0.20 × 0.20 × 0.20 mm

#### Data collection

Rigaku Mercury CCD diffractometer
Absorption correction: multi-scan (*CrystalClear*; Rigaku, 2005 ▶) *T*_min_ = 0.852, *T*_max_ = 1.000
21959 measured reflections
4394 independent reflections
3253 reflections with *I* > 2σ(*I*)
*R*_int_ = 0.049

#### Refinement

*R*[*F* ^2^ > 2σ(*F* ^2^)] = 0.059
*wR*(*F* ^2^) = 0.146
*S* = 1.12
4394 reflections
317 parameters
H atoms treated by a mixture of independent and constrained refinement
Δρ_max_ = 0.13 e Å^−3^
Δρ_min_ = −0.18 e Å^−3^

### 

Data collection: *CrystalClear* (Rigaku, 2005 ▶); cell refinement: *CrystalClear*; data reduction: *CrystalClear*; program(s) used to solve structure: *SHELXS97* (Sheldrick, 2008 ▶); program(s) used to refine structure: *SHELXL97* (Sheldrick, 2008 ▶); molecular graphics: *SHELXTL* (Sheldrick, 2008 ▶); software used to prepare material for publication: *SHELXTL*.

## Supplementary Material

Crystal structure: contains datablock(s) I, global. DOI: 10.1107/S1600536811020757/bh2348sup1.cif

Structure factors: contains datablock(s) I. DOI: 10.1107/S1600536811020757/bh2348Isup2.hkl

Supplementary material file. DOI: 10.1107/S1600536811020757/bh2348Isup3.cml

Additional supplementary materials:  crystallographic information; 3D view; checkCIF report

## Figures and Tables

**Fig. 1.**
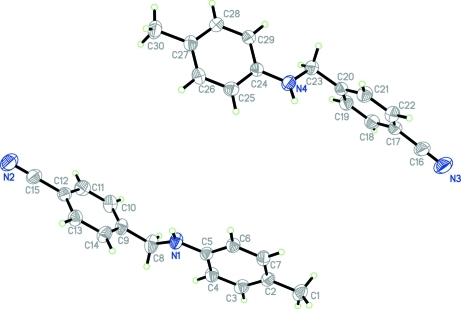
Crystal structure of the title compound with displacement ellipsoids drawn at the 30% probability level.

**Fig. 2.**
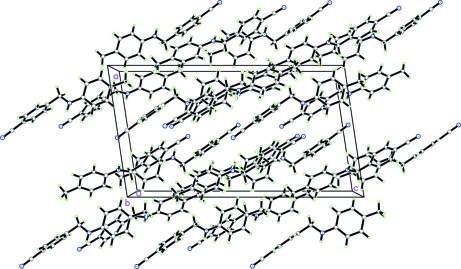
Crystal structure of the title compound with view along the a axis. Intermolecular interactions are shown as dashed lines.

## supplementary crystallographic information

### Comment

We are researching for dielectric-ferroelectric materials. Recent studies have revealed that small molecular compounds which have one or more amidogens probably have this kind of character (Chen *et al.*, 2010). Thus, we want to find aromatic compounds containing amidogens, with the hope to obtain dielectric-ferroelectric properties (Xiong *et al.*, 2002; Zhao *et al.*, 2008). As part of our ongoing studies, we report here the crystal structure of the title compound. The dielectric constant of 4-[(*p*-tolylamino)methyl]benzonitrile as a function of temperature indicates that the permittivity is basically temperature-independent (Wang *et al.*, 2005). After characterization of the dielectric constant, there is no dielectric anomaly observed (Ye *et al.*, 2006). This compound thus should not be a real ferroelectrics or there may be no distinct phase transition occurring within the measured temperature range.

The asymmetric unit of the title compound contains two independent molecules (Fig. 1). The N atom connects two aromatic groups, with bond angles at N1 and N4 being 120.96 (16) and 119.75 (16)°, and the dihedral angle between two benzene rings being 69.1 (2) and 79.9 (2)°, respectively. In the crystal structure, very weak intermolecular interactions link molecules to form one-dimensional ladder propagating along the [010] direction (Fig. 2).

### Experimental

The title compound was obtained by addition of 6.6 g of (*E*)-4-[(*p*-tolylimino)methyl]benzonitrile (30 mmol) to a solution of 4.5 g of sodiumborohydride in THF, in the stoichiometric ratio 1:4. After reacting for 5 h in an ice bath, the mixture was filtered. Good quality single crystals were obtained by slow evaporation of the filtrate over two days.

### Refinement

Amine H atoms H1 and H4 were found in a difference map and refined with free coordinates and *U*_iso_(H) = 0.122 Å^2^. C-bonded H atoms were placed in idealized positions and refined as riding to their carrier atoms, with C—H bond lengths fixed to 0.96 (methyl), 0.97 (methylene) or 0.93 Å (aromatic), and *U*_iso_ fixed at 1.5 or 1.2 times *U*_eq_(carrier C). Methyl H atoms are splitted over two positions, due to free rotation about C2—C1 and C27—C30 bonds. Site occupancies for each group were refined, converging to 0.64 (3)/0.36 (3) for methyl group C1 and 0.66 (3)/0.34 (3) for methyl group C30.

### Crystal data

**Table:**

C_15_H_14_N_2_	*Z* = 8
*M**_r_* = 222.28	*F*(000) = 944
Monoclinic, *P*2_1_/*c*	*D*_x_ = 1.183 Mg m^−^^3^
Hall symbol: -P 2ybc	Mo *K*α radiation, λ = 0.71073 Å
*a* = 12.485 (3) Å	θ = 2.0–27.4°
*b* = 9.112 (3) Å	µ = 0.07 mm^−^^1^
*c* = 22.180 (6) Å	*T* = 293 K
β = 98.537 (6)°	Prism, colourless
*V* = 2495.3 (12) Å^3^	0.20 × 0.20 × 0.20 mm

### Data collection

**Table:**

Rigaku Mercury CCD diffractometer	4394 independent reflections
Radiation source: fine-focus sealed tube	3253 reflections with *I* > 2σ(*I*)
graphite	*R*_int_ = 0.049
Detector resolution: 28.5714 pixels mm^-1^	θ_max_ = 25.0°, θ_min_ = 2.8°
ω scans	*h* = −14→14
Absorption correction: multi-scan (*CrystalClear*; Rigaku, 2005)	*k* = −10→10
*T*_min_ = 0.852, *T*_max_ = 1.000	*l* = −26→26
21959 measured reflections	

### Refinement

**Table:**

Refinement on *F*^2^	Secondary atom site location: difference Fourier map
Least-squares matrix: full	Hydrogen site location: inferred from neighbouring sites
*R*[*F*^2^ > 2σ(*F*^2^)] = 0.059	H atoms treated by a mixture of independent and constrained refinement
*wR*(*F*^2^) = 0.146	*w* = 1/[σ^2^(*F*_o_^2^) + (0.0641*P*)^2^ + 0.2355*P*] where *P* = (*F*_o_^2^ + 2*F*_c_^2^)/3
*S* = 1.12	(Δ/σ)_max_ < 0.001
4394 reflections	Δρ_max_ = 0.13 e Å^−^^3^
317 parameters	Δρ_min_ = −0.18 e Å^−^^3^
0 restraints	Extinction correction: *SHELXL97* (Sheldrick, 2008)
0 constraints	Extinction coefficient: 0.014 (4)
Primary atom site location: structure-invariant direct methods	

### Fractional atomic coordinates and isotropic or equivalent isotropic displacement parameters (Å^2^)

**Table:**

	*x*	*y*	*z*	*U*_iso_*/*U*_eq_	Occ. (<1)
C1	0.9872 (2)	0.3906 (3)	0.28894 (9)	0.0798 (7)	
H1A	1.0443	0.4621	0.2932	0.120*	0.64 (3)
H1B	1.0158	0.2974	0.3038	0.120*	0.64 (3)
H1C	0.9318	0.4211	0.3120	0.120*	0.64 (3)
H1D	1.0630	0.3672	0.2940	0.120*	0.36 (3)
H1E	0.9509	0.3241	0.3127	0.120*	0.36 (3)
H1F	0.9780	0.4894	0.3024	0.120*	0.36 (3)
C2	0.93940 (16)	0.3767 (2)	0.22236 (9)	0.0608 (5)	
C3	0.97256 (17)	0.4659 (2)	0.17850 (9)	0.0665 (5)	
H3A	1.0262	0.5353	0.1904	0.080*	
C4	0.92924 (16)	0.4564 (2)	0.11736 (9)	0.0625 (5)	
H4A	0.9538	0.5189	0.0892	0.075*	
C5	0.84884 (15)	0.3534 (2)	0.09788 (8)	0.0536 (5)	
C6	0.81507 (15)	0.2625 (2)	0.14188 (8)	0.0588 (5)	
H6A	0.7616	0.1928	0.1303	0.071*	
C7	0.85965 (17)	0.2745 (2)	0.20223 (9)	0.0629 (5)	
H7A	0.8355	0.2119	0.2305	0.075*	
C8	0.8210 (2)	0.4451 (2)	−0.00775 (9)	0.0804 (7)	
H8A	0.8976	0.4521	−0.0106	0.097*	
H8B	0.7972	0.5399	0.0052	0.097*	
C9	0.75965 (18)	0.4082 (2)	−0.06918 (8)	0.0626 (5)	
C10	0.6848 (2)	0.5044 (2)	−0.09876 (10)	0.0734 (6)	
H10A	0.6720	0.5929	−0.0802	0.088*	
C11	0.62855 (18)	0.4722 (2)	−0.15532 (10)	0.0727 (6)	
H11A	0.5788	0.5389	−0.1750	0.087*	
C12	0.64640 (15)	0.3395 (2)	−0.18292 (8)	0.0579 (5)	
C13	0.72085 (17)	0.2422 (2)	−0.15366 (9)	0.0655 (6)	
H13A	0.7334	0.1533	−0.1720	0.079*	
C14	0.77679 (19)	0.2767 (2)	−0.09725 (9)	0.0718 (6)	
H14A	0.8269	0.2103	−0.0777	0.086*	
C15	0.58706 (17)	0.3040 (2)	−0.24151 (10)	0.0689 (6)	
C16	0.49515 (19)	0.2565 (3)	0.46950 (11)	0.0853 (7)	
C17	0.42565 (16)	0.2503 (3)	0.41163 (9)	0.0661 (6)	
C18	0.40122 (17)	0.3763 (3)	0.37803 (9)	0.0706 (6)	
H18A	0.4282	0.4663	0.3931	0.085*	
C19	0.33690 (17)	0.3687 (2)	0.32206 (9)	0.0658 (5)	
H19A	0.3208	0.4542	0.2997	0.079*	
C20	0.29561 (15)	0.2362 (2)	0.29845 (8)	0.0567 (5)	
C21	0.31974 (17)	0.1112 (2)	0.33292 (9)	0.0709 (6)	
H21A	0.2922	0.0214	0.3179	0.085*	
C22	0.38393 (18)	0.1168 (3)	0.38921 (10)	0.0744 (6)	
H22A	0.3991	0.0315	0.4119	0.089*	
C23	0.22708 (16)	0.2286 (2)	0.23680 (8)	0.0643 (5)	
H23A	0.1588	0.1809	0.2404	0.077*	
H23B	0.2115	0.3271	0.2214	0.077*	
C24	0.23994 (15)	0.14312 (19)	0.13250 (8)	0.0522 (5)	
C25	0.30602 (16)	0.0980 (2)	0.09069 (9)	0.0622 (5)	
H25A	0.3780	0.0742	0.1042	0.075*	
C26	0.26565 (17)	0.0883 (2)	0.02950 (9)	0.0633 (5)	
H26A	0.3114	0.0572	0.0026	0.076*	
C27	0.15952 (17)	0.1231 (2)	0.00676 (8)	0.0581 (5)	
C28	0.09473 (16)	0.1671 (2)	0.04877 (8)	0.0619 (5)	
H28A	0.0228	0.1908	0.0352	0.074*	
C29	0.13339 (15)	0.1772 (2)	0.11060 (8)	0.0578 (5)	
H29A	0.0872	0.2071	0.1375	0.069*	
C30	0.1177 (2)	0.1150 (3)	−0.06047 (9)	0.0811 (7)	
H30A	0.0513	0.0600	−0.0667	0.122*	0.34 (3)
H30B	0.1704	0.0674	−0.0812	0.122*	0.34 (3)
H30C	0.1046	0.2124	−0.0764	0.122*	0.34 (3)
H30D	0.0445	0.1505	−0.0678	0.122*	0.66 (3)
H30E	0.1195	0.0150	−0.0739	0.122*	0.66 (3)
H30F	0.1623	0.1743	−0.0825	0.122*	0.66 (3)
H1	0.740 (2)	0.293 (3)	0.0310 (11)	0.122*	
H4	0.354 (2)	0.159 (3)	0.2001 (12)	0.122*	
N1	0.80476 (15)	0.33596 (19)	0.03692 (7)	0.0656 (5)	
N2	0.54010 (17)	0.2771 (2)	−0.28871 (9)	0.0944 (7)	
N3	0.55062 (18)	0.2632 (3)	0.51543 (10)	0.1172 (9)	
N4	0.28294 (13)	0.14703 (19)	0.19445 (7)	0.0636 (5)	

### Atomic displacement parameters (Å^2^)

**Table:**

	*U*^11^	*U*^22^	*U*^33^	*U*^12^	*U*^13^	*U*^23^
C1	0.0882 (16)	0.0877 (16)	0.0595 (13)	0.0102 (13)	−0.0023 (12)	−0.0138 (11)
C2	0.0620 (12)	0.0629 (12)	0.0554 (12)	0.0112 (10)	0.0021 (9)	−0.0097 (10)
C3	0.0646 (13)	0.0650 (13)	0.0667 (13)	−0.0053 (10)	−0.0003 (10)	−0.0116 (10)
C4	0.0656 (12)	0.0618 (12)	0.0596 (12)	−0.0097 (10)	0.0081 (10)	−0.0022 (9)
C5	0.0537 (11)	0.0557 (11)	0.0508 (11)	0.0015 (9)	0.0052 (8)	−0.0043 (9)
C6	0.0603 (12)	0.0583 (12)	0.0569 (12)	−0.0062 (9)	0.0058 (9)	−0.0012 (9)
C7	0.0701 (13)	0.0648 (13)	0.0538 (12)	0.0041 (10)	0.0094 (10)	0.0008 (9)
C8	0.1052 (18)	0.0767 (15)	0.0574 (13)	−0.0287 (13)	0.0055 (12)	0.0054 (11)
C9	0.0786 (14)	0.0583 (12)	0.0503 (11)	−0.0117 (11)	0.0077 (10)	0.0082 (9)
C10	0.1007 (17)	0.0545 (12)	0.0661 (14)	0.0003 (12)	0.0161 (13)	0.0035 (10)
C11	0.0747 (14)	0.0653 (14)	0.0772 (15)	0.0119 (11)	0.0087 (12)	0.0165 (11)
C12	0.0556 (11)	0.0634 (13)	0.0544 (12)	−0.0046 (10)	0.0071 (9)	0.0118 (9)
C13	0.0760 (14)	0.0616 (12)	0.0583 (12)	0.0082 (11)	0.0073 (10)	0.0033 (10)
C14	0.0795 (15)	0.0708 (14)	0.0607 (13)	0.0113 (11)	−0.0045 (11)	0.0077 (11)
C15	0.0598 (12)	0.0799 (15)	0.0646 (14)	−0.0101 (11)	0.0016 (11)	0.0125 (11)
C16	0.0597 (13)	0.130 (2)	0.0653 (15)	0.0097 (14)	0.0042 (12)	−0.0150 (14)
C17	0.0509 (11)	0.0955 (16)	0.0517 (12)	−0.0010 (11)	0.0069 (9)	−0.0084 (11)
C18	0.0665 (13)	0.0782 (15)	0.0670 (14)	−0.0130 (11)	0.0096 (11)	−0.0159 (12)
C19	0.0714 (13)	0.0663 (13)	0.0599 (12)	−0.0089 (11)	0.0099 (10)	−0.0017 (10)
C20	0.0530 (11)	0.0674 (13)	0.0511 (11)	−0.0063 (9)	0.0120 (9)	−0.0053 (9)
C21	0.0746 (14)	0.0671 (13)	0.0684 (14)	−0.0136 (11)	0.0024 (11)	−0.0005 (11)
C22	0.0745 (14)	0.0821 (15)	0.0652 (14)	0.0006 (12)	0.0058 (11)	0.0120 (12)
C23	0.0615 (12)	0.0778 (14)	0.0533 (12)	0.0001 (10)	0.0068 (9)	−0.0070 (10)
C24	0.0544 (11)	0.0540 (11)	0.0482 (10)	−0.0010 (9)	0.0079 (8)	−0.0002 (8)
C25	0.0555 (11)	0.0691 (13)	0.0621 (13)	0.0080 (10)	0.0094 (9)	−0.0012 (10)
C26	0.0717 (13)	0.0656 (13)	0.0557 (12)	0.0034 (10)	0.0202 (10)	−0.0024 (9)
C27	0.0667 (12)	0.0562 (11)	0.0515 (11)	−0.0053 (10)	0.0089 (9)	0.0009 (9)
C28	0.0571 (12)	0.0709 (13)	0.0562 (12)	−0.0011 (10)	0.0037 (9)	0.0017 (10)
C29	0.0526 (11)	0.0662 (12)	0.0556 (12)	−0.0003 (9)	0.0108 (9)	−0.0030 (9)
C30	0.0943 (17)	0.0935 (17)	0.0539 (13)	−0.0065 (14)	0.0063 (11)	−0.0026 (11)
N1	0.0718 (11)	0.0724 (12)	0.0503 (10)	−0.0175 (9)	0.0019 (8)	0.0036 (8)
N2	0.0813 (14)	0.1146 (17)	0.0808 (14)	−0.0171 (12)	−0.0097 (11)	0.0060 (12)
N3	0.0791 (15)	0.188 (3)	0.0772 (15)	0.0210 (16)	−0.0122 (12)	−0.0228 (15)
N4	0.0592 (10)	0.0786 (12)	0.0522 (10)	0.0077 (9)	0.0048 (8)	−0.0078 (8)

### Geometric parameters (Å, °)

**Table:**

C1—C2	1.514 (2)	C16—C17	1.440 (3)
C1—H1A	0.9600	C17—C18	1.379 (3)
C1—H1B	0.9600	C17—C22	1.386 (3)
C1—H1C	0.9600	C18—C19	1.376 (3)
C1—H1D	0.9600	C18—H18A	0.9300
C1—H1E	0.9600	C19—C20	1.385 (3)
C1—H1F	0.9600	C19—H19A	0.9300
C2—C3	1.378 (3)	C20—C21	1.379 (3)
C2—C7	1.388 (3)	C20—C23	1.503 (2)
C3—C4	1.386 (3)	C21—C22	1.381 (3)
C3—H3A	0.9300	C21—H21A	0.9300
C4—C5	1.395 (3)	C22—H22A	0.9300
C4—H4A	0.9300	C23—N4	1.454 (2)
C5—N1	1.391 (2)	C23—H23A	0.9700
C5—C6	1.392 (3)	C23—H23B	0.9700
C6—C7	1.376 (2)	C24—C29	1.382 (3)
C6—H6A	0.9300	C24—C25	1.392 (3)
C7—H7A	0.9300	C24—N4	1.400 (2)
C8—N1	1.439 (3)	C25—C26	1.379 (2)
C8—C9	1.499 (3)	C25—H25A	0.9300
C8—H8A	0.9700	C26—C27	1.383 (3)
C8—H8B	0.9700	C26—H26A	0.9300
C9—C10	1.374 (3)	C27—C28	1.381 (3)
C9—C14	1.382 (3)	C27—C30	1.507 (3)
C10—C11	1.376 (3)	C28—C29	1.388 (2)
C10—H10A	0.9300	C28—H28A	0.9300
C11—C12	1.388 (3)	C29—H29A	0.9300
C11—H11A	0.9300	C30—H30A	0.9600
C12—C13	1.375 (3)	C30—H30B	0.9600
C12—C15	1.434 (3)	C30—H30C	0.9600
C13—C14	1.376 (3)	C30—H30D	0.9600
C13—H13A	0.9300	C30—H30E	0.9600
C14—H14A	0.9300	C30—H30F	0.9600
C15—N2	1.148 (2)	N1—H1	0.90 (3)
C16—N3	1.145 (3)	N4—H4	0.89 (3)
			
C2—C1—H1A	109.5	C22—C17—C16	119.9 (2)
C2—C1—H1B	109.5	C19—C18—C17	119.9 (2)
C2—C1—H1C	109.5	C19—C18—H18A	120.1
C2—C1—H1D	109.5	C17—C18—H18A	120.1
C2—C1—H1E	109.5	C18—C19—C20	121.3 (2)
H1D—C1—H1E	109.5	C18—C19—H19A	119.3
C2—C1—H1F	109.5	C20—C19—H19A	119.3
H1D—C1—H1F	109.5	C21—C20—C19	118.14 (19)
H1E—C1—H1F	109.5	C21—C20—C23	120.93 (18)
C3—C2—C7	116.44 (18)	C19—C20—C23	120.93 (18)
C3—C2—C1	121.55 (19)	C20—C21—C22	121.4 (2)
C7—C2—C1	122.0 (2)	C20—C21—H21A	119.3
C2—C3—C4	122.65 (19)	C22—C21—H21A	119.3
C2—C3—H3A	118.7	C21—C22—C17	119.5 (2)
C4—C3—H3A	118.7	C21—C22—H22A	120.2
C3—C4—C5	120.22 (19)	C17—C22—H22A	120.2
C3—C4—H4A	119.9	N4—C23—C20	110.39 (16)
C5—C4—H4A	119.9	N4—C23—H23A	109.6
N1—C5—C6	119.75 (17)	C20—C23—H23A	109.6
N1—C5—C4	122.71 (18)	N4—C23—H23B	109.6
C6—C5—C4	117.51 (17)	C20—C23—H23B	109.6
C7—C6—C5	120.96 (18)	H23A—C23—H23B	108.1
C7—C6—H6A	119.5	C29—C24—C25	117.85 (17)
C5—C6—H6A	119.5	C29—C24—N4	123.25 (17)
C6—C7—C2	122.22 (19)	C25—C24—N4	118.87 (17)
C6—C7—H7A	118.9	C26—C25—C24	120.57 (18)
C2—C7—H7A	118.9	C26—C25—H25A	119.7
N1—C8—C9	111.46 (17)	C24—C25—H25A	119.7
N1—C8—H8A	109.3	C25—C26—C27	122.33 (18)
C9—C8—H8A	109.3	C25—C26—H26A	118.8
N1—C8—H8B	109.3	C27—C26—H26A	118.8
C9—C8—H8B	109.3	C28—C27—C26	116.49 (18)
H8A—C8—H8B	108.0	C28—C27—C30	122.01 (19)
C10—C9—C14	118.53 (19)	C26—C27—C30	121.50 (19)
C10—C9—C8	120.7 (2)	C27—C28—C29	122.23 (18)
C14—C9—C8	120.78 (19)	C27—C28—H28A	118.9
C9—C10—C11	121.2 (2)	C29—C28—H28A	118.9
C9—C10—H10A	119.4	C24—C29—C28	120.53 (18)
C11—C10—H10A	119.4	C24—C29—H29A	119.7
C10—C11—C12	119.63 (19)	C28—C29—H29A	119.7
C10—C11—H11A	120.2	C27—C30—H30A	109.5
C12—C11—H11A	120.2	C27—C30—H30B	109.5
C13—C12—C11	119.65 (19)	C27—C30—H30C	109.5
C13—C12—C15	120.27 (19)	C27—C30—H30D	109.5
C11—C12—C15	120.08 (18)	C27—C30—H30E	109.5
C12—C13—C14	119.9 (2)	H30D—C30—H30E	109.5
C12—C13—H13A	120.0	C27—C30—H30F	109.5
C14—C13—H13A	120.0	H30D—C30—H30F	109.5
C13—C14—C9	121.05 (19)	H30E—C30—H30F	109.5
C13—C14—H14A	119.5	C5—N1—C8	120.96 (16)
C9—C14—H14A	119.5	C5—N1—H1	114.4 (17)
N2—C15—C12	179.1 (2)	C8—N1—H1	114.8 (17)
N3—C16—C17	179.2 (3)	C24—N4—C23	119.75 (16)
C18—C17—C22	119.74 (19)	C24—N4—H4	111.8 (17)
C18—C17—C16	120.3 (2)	C23—N4—H4	114.3 (18)
			
C7—C2—C3—C4	0.4 (3)	C18—C19—C20—C21	0.7 (3)
C1—C2—C3—C4	−179.28 (19)	C18—C19—C20—C23	−178.89 (18)
C2—C3—C4—C5	−0.1 (3)	C19—C20—C21—C22	−0.5 (3)
C3—C4—C5—N1	−178.02 (18)	C23—C20—C21—C22	179.04 (18)
C3—C4—C5—C6	−0.1 (3)	C20—C21—C22—C17	−0.3 (3)
N1—C5—C6—C7	178.03 (18)	C18—C17—C22—C21	1.1 (3)
C4—C5—C6—C7	0.0 (3)	C16—C17—C22—C21	−178.25 (19)
C5—C6—C7—C2	0.2 (3)	C21—C20—C23—N4	−65.8 (2)
C3—C2—C7—C6	−0.4 (3)	C19—C20—C23—N4	113.7 (2)
C1—C2—C7—C6	179.23 (18)	C29—C24—C25—C26	0.1 (3)
N1—C8—C9—C10	122.3 (2)	N4—C24—C25—C26	177.96 (18)
N1—C8—C9—C14	−57.5 (3)	C24—C25—C26—C27	0.4 (3)
C14—C9—C10—C11	−0.6 (3)	C25—C26—C27—C28	−0.7 (3)
C8—C9—C10—C11	179.60 (19)	C25—C26—C27—C30	178.61 (19)
C9—C10—C11—C12	0.8 (3)	C26—C27—C28—C29	0.4 (3)
C10—C11—C12—C13	−0.5 (3)	C30—C27—C28—C29	−178.88 (19)
C10—C11—C12—C15	179.14 (19)	C25—C24—C29—C28	−0.4 (3)
C11—C12—C13—C14	0.2 (3)	N4—C24—C29—C28	−178.12 (17)
C15—C12—C13—C14	−179.46 (18)	C27—C28—C29—C24	0.1 (3)
C12—C13—C14—C9	−0.1 (3)	C6—C5—N1—C8	167.4 (2)
C10—C9—C14—C13	0.3 (3)	C4—C5—N1—C8	−14.7 (3)
C8—C9—C14—C13	−179.9 (2)	C9—C8—N1—C5	−175.91 (19)
C22—C17—C18—C19	−0.9 (3)	C29—C24—N4—C23	−18.5 (3)
C16—C17—C18—C19	178.38 (18)	C25—C24—N4—C23	163.85 (19)
C17—C18—C19—C20	0.1 (3)	C20—C23—N4—C24	−171.62 (16)
